# Clinical outcomes of placenta accreta spectrum in a Tunisian referral maternity unit: A retrospective review

**DOI:** 10.1002/ijgo.71098

**Published:** 2026-05-22

**Authors:** Hannachi Mohammed Amine, Samaali Khaoula, Zangar Salim, Somri Bilel, Hkiri Takwa, Ben Amor Feriel, Ben Aziza Rim, Ben Marzouk Sofiane, Kacem Samia, Malek Monia, Neji Khaled

**Affiliations:** ^1^ Obstetric Department B Maternity and Neonatology Center of Tunis Tunis Tunisia; ^2^ Anesthesiology Department Maternity and Neonatology Center of Tunis Tunis Tunisia; ^3^ Neonatology Department Maternity and Neonatology Center of Tunis Tunis Tunisia

**Keywords:** cesarean hysterectomy, cesarean section, maternal outcomes, neonatal outcomes, placenta accreta spectrum

Placenta accreta spectrum (PAS) can complicate deliveries by unexpected massive bleeding^1^ with a mortality that reaches 7%.^1^ Its incidence has been increasing simultaneously with a rise in delivery rates worldwide.^1^ Antenatal diagnosis has been shown to reduce maternal morbidity and mortality.^2^, ^3^ On the other hand, optimal conditions for delivery require the expertise of a well‐coordinated multidisciplinary team. In the present study, we aimed to report our experience in the management of PAS and its outcomes.

This was a retrospective monocentric study, from January 2015 to March 2025, in a tertiary perinatal center (Department B of the Maternity and Neonatology Center of Tunis, Tunisia). We collected patient medical history, antenatal data, peroperative management, maternal and neonatal outcomes. Data were collected from patients' medical records.

A total of 97 patients were diagnosed with a PAS disorders. The demographic and obstetric characteristics of the 97 patients are summarized in Table 1.

**TABLE 1:** The demographic and obstetric characteristics of the cohort.

	Women with PAS (*n* = 97)
Maternal age (years), median (*from, to*)	35.6 (23,47)
Gravity, median (*from, to*)	3.96 (1,8)
Parity, median (*from, to*)	3.59 (1,6)
Gestational age at prenatal diagnosis (weeks), median (*from, to*)	33.1 (17,39)
Gestational age at delivery (weeks), median (*from, to*)	34 (27,39)
Previous cesarean section, *n* (%)	95 (98%)
0	2 (2%)
1	25 (26%)
2	42 (43%)
3	20 (20%)
≥4	8 (8%)
Placenta previa, *n* (%)	71 (73%)
History of CS and present placenta previa, *n* (%)	71 (73%)
History of curettage, *n* (%)	29 (30%)
History of operative hysteroscopy, *n* (%)	3 (3%)
History of myomectomy, *n* (%)	4 (4%)
History of uterine rupture, *n* (%)	1 (1%)
Histopathologic analysis, *n* (%)	97 (100%)
P. Accreta, *n* (%)	48 (49%)
P. Percreta, *n* (%)	24 (25%)
P. Increta, *n* (%)	25 (26%)

Abbreviations: CS, cesarean section; PAS, placenta accreta spectrum.

Antenatal abdominal and vaginal ultrasound revealed placental lacunae: 60 cases (82%), loss of clear zone: 37 cases (51%), bladder wall interruption: 28 cases (35%), placental bulging: 11 cases (13%) and vascular changes (color Doppler): 37 cases (51%). The sensitivity of ultrasounds was approximately 86%.

A total of 56 placental magnetic resonance imaging (MRI) were performed. The most common MRI markers found were dark placental bands on T2‐weighted imaging (37 cases), placental bulge (19 cases) and disruption between the uterus and the placenta (29 cases).

The median prenatal hospital stay was 14 days. Predelivery management included iron supply, corticoids course, preanesthetic visit, blood transfusions (13 cases) and a consultation with the psychologist.

We performed 45 scheduled cesarean sections. The main indication of emergency delivery was antepartum hemorrhage (27 cases).

We performed cesarean hysterectomy in 82 cases (84.5%). Maternal intra‐ and postpartum morbidity included perioperative transfusion: (62.9%) 61 patients (80.8% of emergency deliveries vs. 42.3% in scheduled deliveries), hypogastric artery ligation: 46 patients (47.4%), and three pelvic packing. The mean postoperative hemoglobin was 9.28 g/dL (5.9–12.3 g/dL). Hematologic complications were thrombocytopenia in 18 cases (18.6%), disseminated intravascular coagulation in six cases (6.1%) and thromboembolic complications in five patients (5.1%). Surgical complications were bladder injury in 19 (19.5%) patients, ureteral injury in five (5.1%), four (4.1%), bowel wounds and five (5.1%) vascular wounds. Nine (9.2%) patients had spinal anesthesia: seven (77.7%) were converted to general anesthesia for hemodynamic instability. Intensive care stay was 3 days ±5 days with 9.56 days of average hospital stay. No maternal deaths were reported.

A conservative approach was attempted in 15 cases, with a success rate not exceeding 53.8%. Secondary complications were identified, including infectious complications: pelvic peritonitis in three cases (3.1%) and hemorrhagic complications: uterine inertia in seven cases (7.2%). Neonatal outcomes included: Prematurity: 58 cases (60%), 22% between 27 and 34 gestational weight (GW), birth weight: 2554 g, Apgar score: six at 1 min and seven at 5 min, intensive care admission: 27 admissions (27.8%), 13.6 days of stay and neonatal mortality: seven cases (7.2%).

To the best of our knowledge, our study is the largest study conducted in Africa.^4^

The combination of prior cesarean section (98%) and placenta previa (73%) emerged as the most commonly identified risk factor. Silver et al. found that the risk of accreta for women with placenta previae and prior cesarean deliveries was 3%, 11%, 40% and 61% for first, second, third, and fourth cesareans, respectively.^2^

Prenatal diagnosis using ultrasound was made in 86% cases. The use of MRI is justified as a second‐line procedure.^1^ Standardized ultrasound criteria are the best choice for antenatal diagnosis of PAS disorders.

Optimal management involves perioperative resuscitation, two experienced seniors in obstetric surgery, a neonatologist, a vascular surgeon, urologist and general surgeon.^4^ In our cohort, surgical complications rate were approximately 40%. Importantly, no deaths were recorded, which may be attributable to the implementation of coordinated multidisciplinary management. Emergency deliveries were associated with high risk of complications.

The median gestational age at delivery was 34 weeks and 6 days in our study, which is consistent with the current literature.^4^ This explains the high prematurity rate, in our case (60%), including 27% admissions in the intensive care unit.

Antenatal diagnosis is an effective mean to reduce maternal morbidity (no maternal deaths were reported in our cohort). Optimal management in a well‐adapted environment (skilled surgeon and experienced intensive care physician with an available blood supply). The radical approach is the preferred management option for known PAS cases.

## AUTHOR CONTRIBUTIONS

Hannachi Mohammed Amine, Ben Marzouk Sofiane, Kacem Samia and Neji Khaled: Conception and design of the study. Samaali Khaoula, Hkiri Takwa, Ben Amor Feriel and Ben Aziza Rim: Data collection. Hannachi Mohammed Amine, Zangar Salim, Ben Amor Feriel and Malek Monia: Data analysis and interpretation. Zangar Salim, Somri Bilel and Ben Aziza Rim: Statistical analysis. Hannachi Mohammed Amine, Samaali Khaoula and Somri Bilel: Manuscript preparation.

## CONFLICT OF INTEREST STATEMENT

The authors declare no conflicts of interest.

## Data Availability

Research data are not shared.
